# P-626. Streptococcus pneumoniae serotype 3 is a leading cause of parapneumonic empyema in northern Israel

**DOI:** 10.1093/ofid/ofaf695.839

**Published:** 2026-01-11

**Authors:** Hikaia Zaidan, Reli Kakun, Sascha Recht, Zohar Steinberg, Yazdani B Shaik-Dasthagirisaheb, Stephen I Pelton, Rotem Lapidot

**Affiliations:** Rambam Healthcare Campus, Haifa, Hefa, Israel; Rambam Healthcare Campus, Haifa, Hefa, Israel; Rambam Healthcare Campus, Haifa, Hefa, Israel; Department of Pediatrics, Carmel Medical Center, Haifa, Israel., AFULA, HaZafon, Israel; Boston Medical Center, Boston, Massachusetts; Boston Medical Center, Boston, Massachusetts; Boston Medical Center, Boston, Massachusetts

## Abstract

**Background:**

Pneumococcal pneumonia is a major health burden among adults and children often leading to complications such as empyema. In Israel, a well-established surveillance system for invasive pneumococcal disease (IPD) has been ongoing since the pre-PCV7 era, but this system does not include empyema cases. Empyema samples often fail to yield results by culture alone, and molecular strategies are essential to identify an accurate and complete picture. Defining distribution of pneumococcal serotypes causing empyema is critical for informing decision makers regarding selection of pneumococcal vaccines.

**Figure 1:**
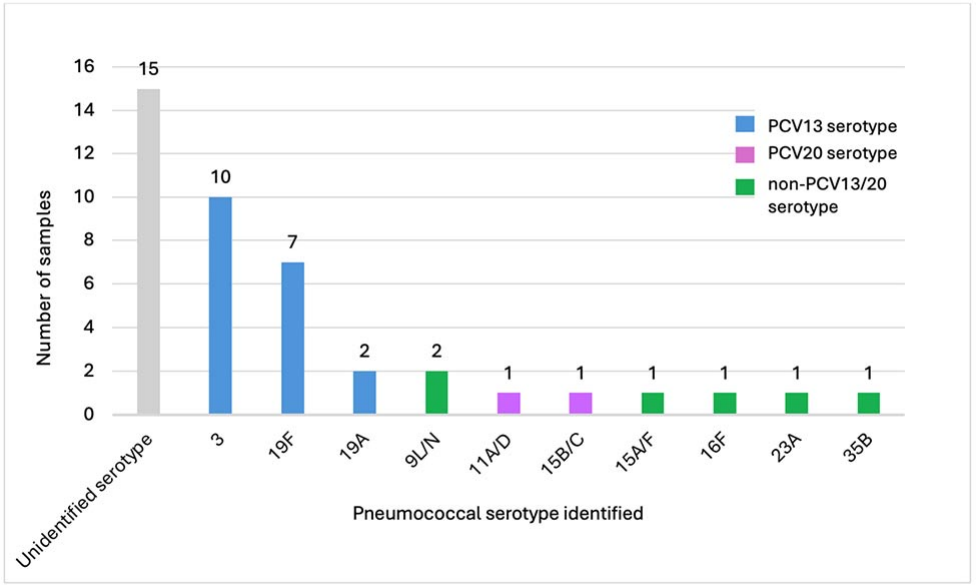
Serotype distribution of pneumococcal empyema in northern Israel

**Methods:**

All pleural fluids from children and adults, collected for clinical indications and sent for microbial detection at 5 medical centers in northern Israel, were included in our study. Samples were shipped with metadata to our research lab at Rambam Healthcare Campus and analyzed for the presence of *S. pneumomiae* (SP) using qPCR. Samples with lytA and piaB probes detected within 35 cycles and with 4 or less cycles between probes were considered positive for SP. Positive samples were subsequently serotyped using qPCR as per the CDC protocol, to identify 25 pneumococcal serogroups/serotypes.

**Results:**

During 2023 we collected 493 pleural fluid samples, 42 (8.5%) samples were positive for SP detection. We identified a serotype in 27 (64.3%) of samples. Serotype 3 was the most common, followed by 19F. Nineteen samples (45%) were identified as PCV13 serotypes despite high coverage in Israeli children (Figure 1).

**Conclusion:**

PCV13 serotypes remain a prevalent cause of pneumococcal empyema in both children and adults in northern Israel. Including empyema samples as part of IPD surveillance systems adds important information to the public health decision process. As PCV20 is introduced into Israel, further studies will be necessary to characterize the impact on serotype epidemiology.

**Disclosures:**

Stephen I. Pelton, MD, CSL Seqirus: Advisor/Consultant|GSK: Grant/Research Support|GSK: Honoraria|Merck Vaccines: Grant/Research Support|Merck Vaccines: Honoraria|Pfizer, Inc.: Grant/Research Support|Pfizer, Inc.: Honoraria|Sanofi: Honoraria|Sanofi: DSMB, Adjudicator for RSV vaccine trial Rotem Lapidot, MD, MSCI, Merck: Advisor/Consultant|Merck: Grant/Research Support|Merck: Honoraria|Pfizer: Advisor/Consultant|Pfizer: Grant/Research Support|Pfizer: Honoraria

